# Type 2 Diabetes and the Use of Real-Time Continuous Glucose Monitoring

**DOI:** 10.1089/dia.2021.0007

**Published:** 2021-03-02

**Authors:** Melanie A. Jackson, Andrew Ahmann, Viral N. Shah

**Affiliations:** ^1^ Division of Endocrinology, Diabetes, and Clinical Nutrition; Oregon Health and Science University, Portland, Oregon, USA.; ^2^ Harold Schnitzer Diabetes Health Center, Oregon Health and Science University, Portland, Oregon, USA.; ^3^ Barbara Davis Center for Diabetes, University of Colorado Anschutz Medical Campus, Aurora, Colorado, USA.

**Keywords:** Type 2 diabetes, Continuous glucose monitor, Glucose monitoring, Glycemic control, HbA1c

## Abstract

The role of continuous glucose monitoring (CGM) in type 1 diabetes (T1D) is well established in improving glycemic control and reducing hypoglycemia. Type 2 diabetes (T2D) is more prevalent than T1D and management of T2D is more heterogeneous, requiring treatment ranging from lifestyle modification to oral medications to intensive insulin therapy. Recent randomized controlled trials in intensively insulin-treated T2D demonstrated the efficacy and safety of real-time CGM (rtCGM) in reducing glycated hemoglobin without increasing hypoglycemia. Although evidence is limited, early studies have indicated a role for rtCGM in selected patients with non-insulin requiring T2D to improve glycemic control and/or reduce hypoglycemia. Based on literature review, we summarized current data on the use of rtCGM in T2D management and provided future research direction to generate more evidence on the utility of CGM in this population.

## Introduction

Diabetes management introduces a significant burden for the patient, who is faced with a wealth of new knowledge at the time of diagnosis. This is later accompanied by ongoing lifestyle modification, multiple medications, and frequent glucose monitoring. As more data have emerged clarifying the importance of glucose monitoring, it has become apparent that treating diabetes without monitoring is comparable to taking a long trip in an automobile without the benefit of a map. Despite glucose meters becoming smaller, faster, and more accurate, while requiring less blood, adherence to self-monitoring of blood glucose (SMBG) is difficult.^1,2^ In addition, capillary glucose measurements do not provide glucose trends, making proactive management unfeasible.

Among the remarkable advances in diabetes technology witnessed by the diabetes community in the past decade, continuous glucose monitoring (CGM) is having the greatest impact. CGM devices have become smaller, more affordable, more accurate, and more user-friendly. With increasing CGM use, most notably in type 1 diabetes (T1D) treatment, and increased awareness of the limitations of HbA1c in the management of diabetes, new CGM-based metrics such as time in range (TIR), time in hypoglycemia, and blood glucose variability (coefficient of variation or CV) have been recommended by an international expert panel as more meaningful targets for diabetes management than HbA1c.^3,4^ From the mean glucose, one can also calculate an estimated HbA1c (eA1c) or the glucose management index, which can be particularly helpful in telemedicine where a laboratory HbA1c value may not be available.

The prevalence of type 2 diabetes (T2D) is higher than T1D, and over time, insulin often becomes necessary to achieve glycemic control.^5–7^ Individualized goals for glycemic control in T1D and T2D are similar, and include TIR greater than 70% of the time, time below range (<70 mg/dL) less than 4%, and glucose variability (CV) ≤36%^4^ (Table 1). Despite numerous advances in therapeutics, many of the patients with T2D are not able to achieve glycemic control.^8^ This may be due to relatively less use of diabetes technology in patients with T2D compared to patients with T1D.

**Table 1.** Recommended Glycemic Targets for Type 2 Diabetes Patients on Continuous Glucose Monitors

Parameter	T2D (%)	T2D with advanced age or significant comorbidities
HbA1c	<7.0	<8.0%
TIR (70–180 mg/dL)	>70	>50%
% Under 70 mg/dL	<4	<1%
% Under 54 mg/dL	<1	To be avoided
Glucose variability (CV)	≤36	≤33%

Adapted from Battelino et al.^4^

CV, coefficient of variation; T2D, type 2 diabetes; TIR, time in range.

Several studies demonstrated improvement in glycemic control and reduction in hypoglycemia with the use of CGM in patients with T1D irrespective of age, sex, educational status, or mode of insulin delivery.^9–15^ Evidence suggests sustained improvement in glycemic control with the use of CGM in early-onset T1D, calling for benefit in starting CGM as early as from the disease onset.^16^ There is a potential role of CGM in T2D as well, with patients facing many of the same obstacles to self-management as those with T1D.^14,17,18^ In addition, CGM has shown characteristics of various pharmacologic agents that aid in their use by differentiating postprandial effects, hypoglycemia risk, and glucose variability.^19,20^

In this review, we searched the literature for published real-time CGM (rtCGM) studies reporting efficacy, safety, quality of life, and lifestyle modifications in patients with T2D. We initiated our search by cross referencing “continuous glucose monitoring” and “type 2 diabetes mellitus” in PubMed. The initial 1678 references were narrowed by adding the search term “real-time” (148 items). Additional refinement included specifying articles representing controlled trials, prospective nonrandomized trials, and previous review articles on the topic, including meta-analyses. Professional guidelines as well as information gathered from professional CGM (blinded) or flash CGM were also reviewed but are cited sparingly to demonstrate principles that could apply to T2D and highlight novel opportunities to expand the use of CGM in T2D. However, the described outcomes are specific to real-time CMG reports.

This is a review of current CGM use and not a comprehensive meta-analysis. Several studies using devices no longer available or not widely used are not included. Importantly, given the high number of individuals with T2D not on insulin therapy, we looked for data that may specifically pertain to this group. Finally, we highlighted some of the limitations of currently available literature and future directions. Randomized controlled trials (RCTs) examining multiple parameters of diabetes care as well as observational studies providing an early look at the role of rtCGM in telemedicine are summarized in Table 2.

**Table 2.** Real-Time Continuous Glucose Monitoring Trials in Patients with Type 2 Diabetes

Author (year)^ref.^	Design	N/Duration	Treatment	Change in HbA1c	Hypoglycemia	QOL/Behavior change	Conclusion
RCTs							
Yoo et al. (2008)^21^	RCT SMBG versus rtCGM	*N* = 65 12 weeks	About 60% on insulin CGM to guide meds/adherence	HbA1c ↓ 1.1% Δ = 0.7%	NR	QOL NR ↓ weight and caloric intake and ↑ exercise	CGM used as a motivational intervention improved HbA1c
Cox et al. (2020)^22^	RCT 1:2 ratio Routine care versus CGM to educate on pp excursions	*N* = 30 2 months active 3-month follow-up	No insulin	HbA1c ↓ 1.3% Decreased medication use	NR	Improved QOL, diabetes distress, and DM knowledge	CGM helps behavioral modification to ↓ pp excursions
Ehrhardt et al. (2011)^23^	RCT rtCGM versus SMBG	*N* = 100 12 weeks	Oral agents ± basal insulin	HbA1c ↓ 1.0% Δ = 0.5%	NR	QOL NR No Δ in weight	First RCT to show benefit with prandial insulin
Beck et al. (2017)^24^	RCT rtCGM versus SMBG Blinded CGM data in SMBG group	*N* = 158 24 weeks	MDI No detailed dietary or exercise effort	HbA1c ↓ 0.8% Δ = 0.3%	Low rate at baseline. No statistical difference	No difference in QOL scales Highly satisfied Perceived less hassle	Best study, but low glucose variability and hypoglycemia at baseline
Telemedicine prospective and observational trials							
Majithia et al. (2020)^25^	Prospective Single-arm study	*N* = 55 4 months	36% on insulin Telemedicine visits with CGM	HbA1c ↓ 1.6% TIR ↑10.2%	No change in glucose less than 70 mg/dL	No QOL study Weight ↓9 lbs	CGM facilitated glucose control in telehealth
Dixon et al. (2020)^26^	Observational study	*N* = 740 90–180 days	Telehealth with CGM support 31% on insulin	HbA1c ↓ 0.85% when starting HbA1c >7.0%	NR	NR	Potential benefit of CGM in telehealth

TIR (70–180 mg/dL).

HbA1c, hemoglobin A1c; CGM, continuous glucose monitoring (refers to rtCGM in this study); MDI, multiple daily insulin dosing; NR, not reported; QOL, quality of life; RCT, randomized controlled trial; rtCGM, real-time CGM; SMBG, self-monitoring of blood glucose.

### Effect of rtCGM on lifestyle modification

Lifestyle modification is recommended as the first step in the management of prediabetes and T2D.^27^ There is evidence in the literature demonstrating that rtCGM use contributes to patient education and assists with behavioral change, indicating it may be an effective teaching tool to modify lifestyle and improve glycemic control.^28,29^

In the first RCT using rtCGM (3 days at a time for 3 months), Yoo et al. randomized 65 patients with poorly controlled T2D on oral and/or insulin therapy to either rtCGM or SMBG. They demonstrated a 0.7% greater HbA1c reduction in the rtCGM intervention group compared to a group randomized to SMBG alone, as well as changes in weight, exercise, and postprandial glucose.^21^ Looking at rtCGM incorporation in diabetes education, Lee et al. sequentially enrolled T2D patients receiving initial diabetes education into groups of pattern management guided by Guardian rtCGM or standard diabetes education as a control.^30^ The participants were on a mix of therapies, including insulin and non-insulin medications. The rtCGM group showed improvement in classic lifestyle factors of diet, exercise, and self-management concepts. The overall self-care behavior score was significantly higher in the rtCGM group 3 and 6 months after the education was completed, showing a role for rtCGM in developing habits that are helpful for diabetes management in a standard T2D population.

Cox et al. created a model using education and lifestyle interventions to minimize glucose excursions and employed rtCGM (Dexcom Platinum G4) to educate patients on the effects of eating and activity choices previously demonstrated to reduce glycemic variability.^22^ They found not only an improvement in HbA1c but also a remarkable improvement in self-reported diabetes knowledge in those using rtCGM. Taylor et al. explored the educational potential of rtCGM in a pilot study where 20 obese patients with T2D were randomized to either professional CGM or rtCGM, in addition to standard lifestyle modification over 12 weeks.^31^ They saw reductions in HbA1c and body weight in both groups without any statistical difference between the two groups. However, authors reported a 40% greater reduction in blood glucose-lowering medication in the rtCGM group compared to the control group. In a systematic review by the same author that included 5542 participants from 11 studies (eight RCTs and three observational studies), CGM use (either professional or rtCGM) was associated with decreased body weight, decreased caloric intake, higher adherence to eating plans, and increased physical activity compared to SMBG.^32^

These studies demonstrate rtCGM has the potential to improve lifestyle changes and adherence to treatment in patients with T2D. However, most of the studies were of shorter duration with small sample sizes limiting the evidence of rtCGM as an adjunct to lifestyle management in patients with T2D.

### Improvement in glucose control with rtCGM

In an early RCT examining CGM use by Garg et al., 91 insulin-requiring patients with diabetes (75 with T1D and 16 with T2D) were randomized to receive 3 days of rtCGM (STS, Dexcom) or masked CGM (control).^33^ When compared with control subjects, the CGM group spent 21% less time in hypoglycemia (<55 mg/dL), 23% less time in hyperglycemia (≥240 mg/dL), and 26% more time in the target (81–140 mg/dL) glucose range. This was the first study to demonstrate the feasibility and safety of rtCGM in insulin-treated diabetes. The data for those with T2D were not separately analyzed and thus, potential glycemic benefit in this group was not assessed. Similarly, in a small observational study of 140 patients with diabetes (109 with T1D, 24 with T2D on insulin, and 7 with T2D on non-insulin therapies), rtCGM (STS Dexcom) was associated with a significant reduction in HbA1c by 0.4% in both T1D and T2D over 3 months compared to baseline.^34^ Those individuals with T2D on non-insulin medications did not have a significant HbA1c reduction, probably due to small sample size. Similarly, in the GLADIS (Glucose Awareness in Diabetes Study) clinical trial, New et al. randomized 100 patients with insulin-treated diabetes (including 19 with T2D) to either rtCGM (Freestyle Navigator) or SMBG.^35^ With the small sample size, there was no improvement detected in HbA1c in the rtCGM group compared to SMBG.

An RCT by Ehrhardt et al. randomized 100 patients with T2D on various therapies, excluding prandial insulin to either usual care or four cycles of rtCGM (Dexcom SEVEN, 2 weeks on/1 week off) over 12 weeks.^23^ After 12 weeks, all subjects were followed by their primary care providers for an additional 40 weeks. There was significant improvement in HbA1c by 0.5% in the rtCGM group compared to control over 12 weeks, and the glycemic benefits seen with rtCGM use persisted over 40 weeks.^36^

Earlier systematic reviews and meta-analyses of CGM studies (including both rtCGM and professional CGM) suggested benefits of CGM in improving glycemic control, especially in insulin-treated patients with T2D.^28,37^ The DiaMonD (Multiple Daily Injections and CGM in Diabetes**)** trial was a larger study that randomized 158 individuals with T2D using multiple daily insulin (MDI) to either rtCGM (Dexcom G4 with 505 software) or SMBG with the goal of further evaluating the effectiveness of rtCGM in individuals with T2D.^24^ The study design included minimal patient contact, designed to replicate a real-world scenario. Despite this, there was significant reduction in HbA1c by 1% at 12 weeks and 0.8% at 24 weeks. The control and intervention groups both showed improvement in HbA1c at 12 weeks, but there remained a significant difference between the two, with the rtCGM group showing an additional 0.3% decrease (95% confidence interval 0.6%–0.1%, *P* = 0.005). Although this difference is of debatable clinical significance, there were also advantages in the proportion of subjects with a 10% reduction in HbA1c (57% vs. 35%, *P* = 0.016), proportion with ≥1% reduction in HbA1c (53% vs. 33%, *P* = 0.034), and proportion with ≥0.5% reduction HbA1c (79% vs. 51%, *P* = 0.002) in the rtCGM group compared to SMBG group. TIR was also higher in the rtCGM group.

A recent meta-analysis looked exclusively at RCTs specifying individuals with T2D and examined HbA1c as an outcome.^38^ They identified five studies that included a total of 382 patients and found a significant improvement in HbA1c (pooled mean difference of 0.25%) with the use of rtCGM compared to SMBG, providing additional evidence of HbA1c change with rtCGM use.

The role of rtCGM in telemedicine to improve HbA1c was studied by Majithia et al., who used Dexcom G6 sensors as part of a clinical model to evaluate patient response to medical interventions. HbA1c from an initial 10-day run-in period was compared to HbA1c in a 10-day period later in the study. While there was no separate control group in this study, their interventions showed an improvement of 1.6% in HbA1c, from a mean of 8.9% at baseline to 7.3% at follow-up (*P* < 0.001).^25^

Dixon et al. studied a telehealth diabetes clinic model utilizing rtCGM (Dexcom G5 or G6) in 740 participants enrolled across 21 states, with groups stratified by starting HbA1c. All groups with HbA1c >7% saw a significant change ranging from 0.2% ± 0.8% with initial HbA1c values 7.0%–7.9% to 2.3% ± 1.0% for those with baseline Hba1c >9.0% (*P* < 0.001), while those with HbA1c <7% maintained this level of glycemic control.^26^ In conclusion, there is mounting evidence that rtCGM can improve glycemic control in insulin-treated patients with T2D, in addition to other benefits.

### Effect of rtCGM on hypoglycemia

Optimal glycemic control is necessary to prevent microvascular complications in patients with diabetes. Hypoglycemia, however, remains a major obstacle to achieving this. Severe hypoglycemia is a serious acute complication of diabetes that affects many more patients with T2D than we generally recognize.^39–42^ Patients with elevated blood glucose are not protected from hypoglycemia, as studies seeking to identify which patients have elevated risk of hypoglycemia found that blood glucose variability was a strong predictor.^43,44^ In a study of 108 patients with T2D on a mix of insulin and non-insulin agents, Gehlaut et al. found that 49% of individuals experienced hypoglycemia at least once during a 5-week period of professional CGM monitoring (Medtronic iPro), and that many of these episodes were asymptomatic.^40^ In addition, 21% of patients had blood glucose levels of 50 mg/dL or lower. Several studies have suggested that SMBG underreports hypoglycemia in patients with T2D, and it is thought that CGM would more effectively detect hypoglycemia.^45–47^

These studies shine light on the important and perhaps underrecognized issue of hypoglycemia in patients with T2D. The main question, however, is whether rtCGM is effective in reducing hypoglycemia in this population.

In the largest prospective trial of subjects with T2D treated with MDI, Beck et al. did not find a statistically significant reduction in hypoglycemia less than 70 mg/dL with use of rtCGM, although with a rate of only 11 min per day of hypoglycemia, the study was not powered to detect a difference.^24^ There was a nonsignificant reduction in hypoglycemia from a median of 11 minutes per day to 4 minutes per day in the rtCGM group, while the SMBG group experienced a median of 12 minutes per day in hypoglycemia both at baseline and at the 24-week mark.

Despite the very high prevalence of hypoglycemia in patients with T2D, there is no RCT using rtCGM with the primary objective of hypoglycemia reduction. T2D patients with hypoglycemia unawareness or with a greater percentage of hypoglycemia at baseline are likely to benefit from rtCGM; however, further dedicated studies are necessary to establish evidence of rtCGM utility in hypoglycemia prevention or reduction.

### Glucose variability outcomes with rtCGM

HbA1c has been a longstanding measure of adequate glycemic control in individuals with T2D, although it has a known limitation of incompletely describing glycemic variability. There is growing evidence that glycemic variability predicts chronic complications. Cross-sectional studies have found associations between glycemic variability and microvascular complications such as diabetic retinopathy, painful diabetic neuropathy, and cardiovascular autonomic neuropathy in patients with T2D.^24,48–50^ Moreover, the need for cardiac revascularization in patients observed following STEMI was shown to be greater in those with higher glycemic variability.^51^

Although studies of rtCGM in patients with T1D have demonstrated reduced variability, it has not been adequately studied in T2D.^10,52^ In a clinical trial that used glucose variability as a secondary outcome, the baseline rate of variability was quite low (31%) and no significant difference was noted compared to SMBG.^53^ At present, it has not been clearly demonstrated that reducing glycemic variability would reduce diabetes complications or cardiovascular events.

### Patient satisfaction and quality of life with rtCGM

T2D is associated with reduced health-related quality of life.^54^ The high work load of SMBG and predicting blood glucose levels are a frequent cause of concern. A survey-based study by Runge et al. found that wearing an rtCGM and the associated increased awareness of TIR reduced stress experienced by those with diabetes.^55^ Vigersky et al. found no difference in diabetes distress in their study on intermittent CGM use, although the participants in the rtCGM group were requested to perform fingersticks at the same frequency as the SMBG group, negating any difference in work load.^36^ In a study by Beck et al., the use of rtCGM (Dexcom G4 with 505 software) in T2D patients on MDI showed a high level of satisfaction with CGM (4.3 with a scale of 1–5) and a low “hassle” subscore.^24^ Other evaluations of patient-reported outcomes have found high levels of use, good satisfaction, and a better understanding of their glucose patterns, among other benefits.^56,57^

Quality of life remains a measure that is less frequently reported. There is a logical trend toward improved quality of life in individuals who have more health knowledge and less hassle, but currently available evidence is insufficient to fully understand the effect of rtCGM on patient satisfaction and quality of life.

### Recommendation for the use of rtCGM in T2D by the professional societies

All three major U.S. diabetes professional societies have provided evidence-based guidance on the use of CGM for patients with T2D.^58–61^ All professional organizations have recommended CGM use in selected patients with T2D to improve glycemic control or reduce hypoglycemia, while acknowledging limited and low-quality evidence, especially non-insulin-treated T2D. A summary of their recommendations is provided in Table 3.

**Table 3.** Professional Society Recommendations for Continuous Glucose Monitoring Use in the Management of Type 2 Diabetes

Professional society (reference)	Recommendations
ADA^55^	When used properly, real-time continuous glucose monitors in conjunction with multiple daily injections and continuous subcutaneous insulin infusion [A], and other forms of insulin therapy [C] are a useful tool to lower and/or maintain A1C levels and/or reduce hypoglycemia in adults and youth with diabetes. Use of professional CGM and/or intermittent real-time or intermittently scanned CGM can be helpful in identifying and correcting patterns of hyperglycemia and hypoglycemia, and improving A1C levels in people with diabetes on noninsulin, as well as basal insulin regimens. [C]
AACE^56^	CGM devices should be considered for patients with T1D and T2D who are on intensive insulin therapy to improve HbA1c levels and reduce hypoglycemia (Grade B), early reports suggest that even patients not taking insulin may benefit from CGM (Grade D).
The Endocrine Society^57,58^	We suggest short-term, intermittent rtCGM use in adult patients with T2DM (not on prandial insulin), who have A1c levels >7% and are willing and able to use the device. (2|⊕⊕○○)

ADA level A evidence—high-level, clear evidence from well conducted, generalizable RCT, ADA level C evidence—supportive evidence from well-conducted studies. AACE grade B evidence is intermediate level, while D means not evidence based. Endocrine society level of evidence 2|⊕⊕ means weak, low-quality evidence.

AACE, American Association of Clinical Endocrinologists; ADA, American Diabetes Association; T1D, type 1 diabetes.

### Other considerations and future directions

The use of rtCGM for those with T2D will continue to evolve. The trials done thus far are small, but they add valuable information, showing a clear potential benefit of using rtCGM to more effectively educate patients and assist them in improving their diabetes management. Practitioners involved in providing CGM to patients will seek more guidance than is presently available to make educated choices about which patients will benefit most from this technology, and how to optimize its use in those individuals. Although an early estimate of cost-effectiveness has been favorable at $33,039 per quality-adjusted life year, insurers are likely to seek more outcomes data and reduced cost to apply to the widest spectrum of patients with T2D.^62^ However, cost-effectiveness may be demonstrated for a subset of the T2D population, particularly if they have significant hypoglycemia or high glycemic variability. So far, no study in T2D has selected patients with documented hypoglycemic unawareness or a history of severe hypoglycemic episodes. It will also be important to study patients with advanced age and with comorbidities that may increase their risk of hypoglycemia.

Telemedicine has greatly expanded during the COVID-19 pandemic, and the role of CGM in communicating patient data to providers has been crucial. This will likely remain an important tool in maximizing patient access options and improving communication with providers.^25,26,56^

Another developing application of rtCGM relevant to both T1D and T2D is use in the inpatient setting, where frequent monitoring with remote display may show significant advantages in nursing demands, more dynamic therapeutic adjustments, and reduced hypoglycemia.^63–67^ This use requires further research, including cost-benefit analysis, but holds great promise. Researchers and manufacturers have made efforts to improve the human interface in CGM technology. However, future studies should include more patient education on how to most effectively use the information provided by CGM to modify behavior, empowering patients to optimize their self-management skills.

## Conclusions

The development of CGM has had a profound impact on the field of diabetes. There is real potential for rtCGM to improve glycemic outcomes and prevent diabetes complications in patients with T2D. Current evidence is promising, but the majority of trials looking at the role of CGM with T2D management have selected those on intensive insulin therapies, leaving out a large number of T2D patients. Studies have suggested a role for rtCGM as a teaching tool to improve lifestyle management, which would be helpful even in early T2D or prediabetes. These studies have been small, however, and their data often inconclusive. This exciting new field would benefit from large RCTs confirming usefulness of rtCGM for lifestyle improvement and diabetes self-management, which remain the mainstay of diabetes care.
